# Relative risk of functional dyspepsia in patients with sleep disturbance: a population-based cohort study

**DOI:** 10.1038/s41598-021-98169-4

**Published:** 2021-09-20

**Authors:** Hsu-Han Su, Fung-Chang Sung, Kai-Liang Kao, Shu-Chin Chen, Chen-Ju Lin, Shu-I Wu, Cheng-Li Lin, Robert Stewart, Yi-Shin Chen

**Affiliations:** 1 Department of Psychiatry, Hsin-Chu Mackay Memorial Hospital, Hsin-Chu, Taiwan; 2 Department of Health Services Administration, College of Public Health, China Medical University, Taichung, Taiwan; 3 Management Office for Health Data, China Medical University Hospital, Taichung, Taiwan; 4 Department of Food Nutrition and Health Biotechnology, Asia University, Taichung, Taiwan; 5 Far Eastern Memorial Hospital, Department of Pediatrics, Taipei, Taiwan; 6 Suicide Prevention Center, Section of Psychiatry, Mackay Memorial Hospital, Taipei, Taiwan; 7 Department of Medicine, Mackay Medical College, Taipei, Taiwan; 8 Management Office for Health Data, China, Medical University Hospital, Taichung, Taiwan; 9 King’s College London (Institute of Psychiatry, Psychology and Neuroscience), London, UK; 10 South London and Maudsley NHS Foundation Trust, London, UK; 11 Department of Computer Science, National Tsing Hua University, Hsinchu, Taiwan

**Keywords:** Diseases, Health care

## Abstract

Increased prevalence of sleep disorders has been found in patients with functional dyspepsia; however, direction of causality remains unclear. Our aim was to compare the risk of incident functional dyspepsia between patients with and without sleep disturbance from a large population-based sample. Utilizing a nation-wide health insurance administrative dataset, we assembled an 11-year historic cohort study to compare subsequent incidence of diagnosed functional dyspepsia between adult patients with any diagnosis of sleep disturbance and age- and gender-matched controls. Hazard ratios adjusted for other relevant comorbidities and medications were calculated using Cox regression models. 45,310 patients with sleep disorder and 90,620 controls were compared. Patients with sleep apnea had a 3.3-fold (95% confidence interval: 2.82 ~ 3.89) increased hazard of functional dyspepsia compared with controls. This increased risk persisted regardless of previously diagnosed depression coexisted. Sleep disturbance was associated with an increased risk of subsequent functional dyspepsia. Potential mechanisms are discussed.

## Introduction

It is estimated that over 20% of the general population have sleep problems, and more than 8% suffer from chronic sleep disturbance^1,2^. Sleep disturbance can be categorized into insomnia, or sleep disorders such as intrinsic or extrinsic dyssomnias, and parasomnias^3^ and have been found to be associated with a wide range of impairments in physical and mental health, as well as in social and emotional function^1,4^. Associations described with physical disorders have included diabetes^5^, cardiovascular diseases^4^, and impaired liver function^6,7^, although these have focused on moderate or severe sleep apnea rather than wider sleep disturbance.

Functional dyspepsia, affecting about 20% of the population^8^, is a common gastrointestinal disorder with symptoms of bothersome postprandial fullness, early satiation, epigastric pain, or gastric burning for the last 3 months without evidence of metabolic or structural diseases^9^. Previous research has suggested disrupted sleep quality in patients with dyspepsia compared to healthy controls, with published odds ratios ranging from 1.7 to 3.4^10–14^. However, some studies have not found this association^15^ and others have found sleep apnea to be associated with irritable bowel syndrome rather than dyspepsia^16–18^. In addition, most research in this area has involved cross-sectional or case–control designs, providing limited information on the direction of causality.

We therefore aimed to compare the prospective risk of functional dyspepsia between patients with and without diagnosed sleep disturbance using a nationwide database of health service utilization. Interactions of depression and incident dyspepsia were also investigated to clarify further the association of interest.

## Results

### Comparisons of characteristics in patients with or without sleep disturbances

Table 1 compares demographic characteristics, comorbidities, and medications between the 36,044 patients identified with sleep disturbance (758 apnea, 35,286 non-apnea) and the 66,768 matched controls. The mean age of the sleep disturbance cohort was 52.2 years, and that of the control cohort was 52.0 years. Among those with sleep disturbance, the mean age of those in the apnea subgroup were slightly younger, with more than half under 50 years of age. Males predominated in the apnea subgroup, and females in the non-apnea subgroup. Compared with the control cohort, comorbidities and medications of interest were more common in the sleep disturbance cohort. Mean follow-up durations were 9.76, 9.16 and 9.71 years for the apnea subgroup, non-apnea subgroup, and control cohorts, respectively.

**Table 1:** Comparison of cohort characteristics.

	Sleep disturbance						Control		p-value
	Apnea subgroup (N = 948)		Non-apnea subgroup (N = 44,362)		Total (N = 45,310)		(N = 90,620)		
	n	%	n	%	n	%	n	%	
**Age, year**									0.99
≤ 34	166	17.5	7017	15.8	7183	15.9	14,366	15.9	
35–49	365	38.5	14,078	31.7	14,443	31.9	28,886	31.9	
50–64	255	26.9	12,054	27.2	12,309	27.2	24,618	27.2	
≥ 65	162	17.1	11,213	25.3	11,375	25.1	22,750	25.1	
Mean (SD) ^#^	49.4	14.5	52.3	16.0	52.2	16.0	52.0	16.1	0.03
**Gender**									0.99
Women	365	38.5	28,120	63.4	28,485	62.9	56,970	62.9	
Men	583	61.5	16,242	36.6	16,825	37.1	33,650	37.1	
**Comorbidity**									
Diabetes	69	7.28	3294	7.43	3363	7.42	4372	4.82	< 0.001
Hypertension	390	41.1	17,341	39.1	17,731	39.1	21,182	23.4	< 0.001
Hyperlipidemia	252	26.6	9092	20.5	9344	20.6	9159	10.1	< 0.001
Anxiety	47	4.96	2345	5.29	2392	5.28	467	0.52	< 0.001
Depression	57	6.01	2844	6.41	2901	6.40	512	0.56	< 0.001
Peptic ulcer disease	190	20.0	9076	20.5	9266	20.5	7126	7.86	< 0.001
Stroke	29	3.06	1639	3.69	1668	3.68	2347	2.59	< 0.001
Ischemic heart disease	219	23.1	9364	21.1	9583	21.2	8335	9.20	< 0.001
Obesity	20	2.11	294	0.66	314	0.69	272	0.30	< 0.001
Irritable bowel syndrome	25	2.64	1169	3.76	1694	3.74	883	0.97	< 0.001
Cholecystitis	3	0.32	115	0.26	118	0.26	87	0.10	< 0.001
Cholelithiasis and other disorders of biliary tract	19	2.00	943	2.13	962	2.12	823	0.91	< 0.001
Alcoholism	26	2.74	1071	2.41	1097	2.42	565	0.62	< 0.001
Heart failure	25	2.64	1046	2.36	1071	2.36	1148	1.27	< 0.001
Atrial fibrillation	7	0.74	257	0.58	264	0.58	325	0.36	< 0.001
Chronic kidney disease	95	10.0	3997	9.01	4124	4.55	4092	9.03	< 0.001
Chronic obstructive pulmonary diseases	118	12.5	4416	9.95	4534	10.0	4001	4.42	< 0.001
Asthma	74	7.81	2552	5.75	2626	5.80	2081	2.30	< 0.001
Fatigue	90	9.49	4637	10.5	4727	10.3	4520	4.99	< 0.001
**Medication**									
Benzodiazepine	901	95.0	43,376	97.8	44,277	97.7	65,493	72.3	< 0.001
Zolpidem	562	59.3	27,375	61.7	27,937	61.7	9282	10.2	< 0.001
Aspirin	238	25.1	9908	22.3	10,146	22.4	11,487	12.7	< 0.001
NSAIDs	846	89.2	39,365	88.7	40,211	88.9	61,829	68.2	< 0.001
Steroids	454	47.9	18,764	42.3	19,218	42.4	23,860	26.63	< 0.001
Proton pump inhibitors	58	6.12	2274	5.13	2332	5.15	2280	2.52	< 0.001
H2-receptor antagonists	355	37.5	18,161	40.9	18,516	40.9	22,153	24.5	< 0.001

Chi-square test compared to total SD.

^#^T-test.

### Cumulative incidences of dyspepsia in patients with or without sleep disturbances

**Figure 1:**
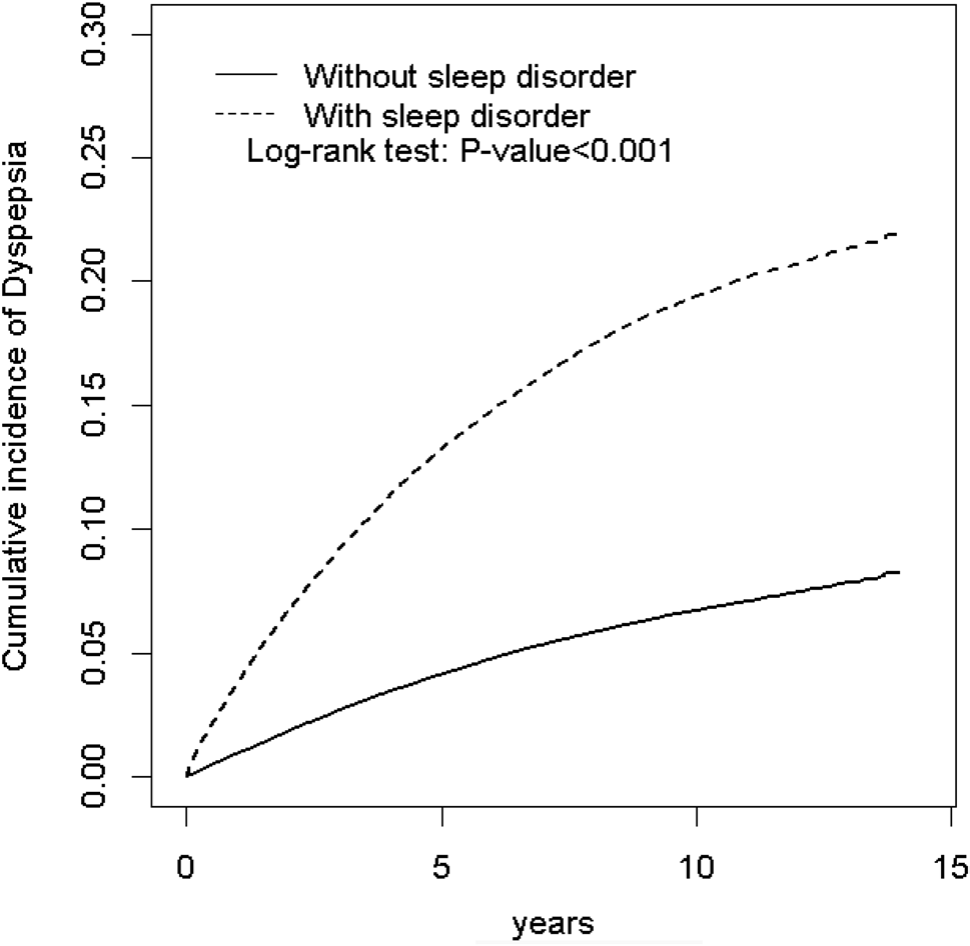
Kaplan-Meir method determined cumulative incidence of Dyspepsia compared between sleep disorder cohorts and comparisons without sleep disorder.

As indicated in Fig. 1, the cumulative incidence of dyspepsia in patients with sleep disturbance was approximately 10.5% higher than that in matched controls (log-rank test p < 0.001). The overall incidence density of dyspepsia was significantly higher in both the apnea and non-apnea subgroups compared to the control cohort (18.9, 210, and 6.73 instances per 1000 person-years, respectively (Table 2)).

**Table 2:** Comparisons of incidence densities and hazard ratios of dyspepsia in exposed and control cohorts.

	Control		Apnea subgroup		Crude HR (95% CI)	Adjusted HR^†^	Non-apnea subgroup		Crude HR (95% CI)	Adjusted HR^†^
	Case	Rate^#^	Case	Rate^#^		(95% CI)	Case	Rate^#^		(95% CI)
All	5919	6.73	175	18.9	2.80 (2.41,3.26)	3.31 (2.82, 3.89)	8532	21.0	3.08 (2.98,9.18)	3.98 (3.83,4.14)
**Age**										
≤ 34	589	4.04	24	14.2	3.50 (2.33, 5.26)	3.72 (2.38, 5.80)	1201	17.3	4.24 (3.84, 4.68)	5.35 (4.76, 6.02)
35–49	1560	5.08	62	16.7	3.25 (2.52, 4.19)	3.27 (2.46, 4.34)	2638	19.1	3.71 (3.48, 3.95)	4.82 (4.47, 5.20)
50–64	1891	7.54	47	18.6	2.43 (1.82,3.25)	2.45 (1.80,3.33)	2375	21.0	2.74 (2.58,2.91)	3.68 (3.42,3.96)
> 64	1879	10.7	42	32.3	3.04 (2.24,4.13)	4.01 (2.90,5.54)	2318	27.1	2.52 (2.37,2.68)	3.36 (3.12,3.61)
**Gender**										
Women	3889	6.89	81	23.3	3.35 (2.69,4.18)	3.77 (2.98,4.76)	5534	20.9	3.00 (2.88,3.13)	3.99 (3.80,4.19)
Men	2030	6.44	94	16.3	2.54 (2.06,3.12)	3.14 (2.51, 3.93)	2998	21.2	3.23 (3.05,3.41)	3.99 (3.73,4.27)
**Comorbidity**^‡^										
None	2890	4.88	47	14.9	3.05 (2.29,4.07)	4.09 (3.04,5.50)	2353	15.8	3.23 (3.06,3.41)	4.52 (4.24,4.83)
Diabetes	311	9.65	18	32.0	3.37 (2.09, 5.42)	4.53 (2.67, 7.67)	610	23.5	2.48 (2.16, 2.84)	3.67 (3.14, 4.28)
Hypertension	1839	10.2	85	23.7	2.34 (1.88, 2.90)	3.59 (2.84, 4.54)	3535	23.9	2.34 (2.21, 2.48)	3.59 (3.37, 3.83)
Hyperlipidemia	895	10.5	52	21.8	2.08 (1.58, 2.76)	2.79 (2.05, 3.80)	2056	25.3	2.40 (2.22, 2.59)	3.77 (3.44, 4.11)
Anxiety	47	11.3	14	34.5	3.05 (1.68, 5.55)	7.19 (3.12, 16.6)	564	27.9	2.46 (1.83, 3.31)	4.15 (3.04, 5.65)
Depression	61	14.9	10	17.8	1.18 (0.60, 2.33)	1.72 (0.76, 3.90)	633	25.2	1.76 (1.35, 2.29)	2.73 (2.08, 3.61)
Peptic ulcer disease	828	14.1	40	23.2	1.67 (1.22, 2.30)	2.67 (1.89, 3.77)	2239	30.0	2.12 (1.96, 2.30)	3.35 (3.06, 3.67)
Stroke	159	11.8	6	25.9	2.44 (1.08, 5.51)	2.96 (1.24, 7.08)	269	23.3	2.96 (1.24, 7.08)	2.85 (2.28, 3.55)
Ischemic heart disease	843	12.7	46	22.9	1.85 (1.37, 2.49)	2.78 (2.02, 3.83)	2186	28.6	2.27 (2.09, 2.45)	3.50 (3.20, 3.82)
Obesity	24	9.21	3	15.2	1.63 (0.49, 5.41)	2.11 (0.42, 10.6)	48	17.5	1.88 (1.15, 3.06)	3.06 (1.70, 5.51)
Irritable bowel syndrome	132	18.0	8	35.1	2.01 (0.99, 4.11)	4.19 (1.72, 10.2)	511	38.1	2.10 (1.74, 2.54)	3.21 (2.60, 3.96)
Cholecystitis	9	12.2	0	0.00	–	–	30	32.0	2.65 (1.26, 2.57)	7.23 (2.62, 20.0)
Cholelithiasis and other disorders of biliary tract	103	15.6	9	60.9	3.76 (1.90, 7.44)	7.02 (2.82, 17.5)	223	29.0	1.87 (1.48, 2.36)	2.99 (2.26, 3.98)
Alcoholism	41	9.00	7	31.3	3.49 (1.57, 7.78)	3.27 (1.07, 9.94)	187	21.7	2.41 (1.72, 3.38)	4.29 (2.92, 6.30)
Heart failure	95	14.6	4	20.5	1.49 (0.55, 4.05)	2.35 (0.79, 6.96)	222	32.4	2.30 (1.81, 2.92)	3.53 (2.72, 4.58)
Atrial fibrillation	19	9.08	0	0.00	–	–	44	26.4	2.93 (1.71, 5.03)	3.83 (2.07, 7.10)
Chronic kidney disease	448	14.5	21	24.2	1.71 (1.11, 2.66)	2.85 (1.77, 4.59)	921	29.2	2.05 (1.83, 2.29)	3.14 (2.77, 3.57)
Chronic obstructive pulmonary diseases	372	12.7	21	19.9	1.63 (1.05, 2.53)	2.76 (1.71, 4.45)	1033	30.8	2.46 (2.18, 2.77)	3.91 (3.43, 4.46)
Asthma	217	13.4	12	18.0	1.37 (0.77, 2.45)	2.32 (1.24, 4.34)	605	29.8	2.24 (1.92, 2.62)	3.66 (3.08, 4.35)
Fatigue	109	2.16	7	7.02	3.35 (1.56, 7.20)	5.11 (2.14, 12.2)	280	5.46	2.56 (2.05, 3.20)	2.54 (1.92, 3.35)
**Benzodiazepine**										
No	1357	5.62	3	6.01	1.09 (0.35, 3.38)	1.12 (0.36, 3.50)	279	32.5	5.66 (4.98, 6.44)	6.68 (5.83, 7.66)
Yes	4562	7.15	172	19.7	2.74 (2.35, 3.19)	3.43 (2.91, 4.04)	8253	20.8	2.87 (2.77, 2.97)	3.86 (3.70, 4.02)
**Zolpidem**										
No	5561	7.03	89	25.0	3.52 (2.86, 4.34)	3.29 (2.66, 4.07)	4908	35.9	4.90 (4.72, 5.10)	4.00 (3.83, 4.17)
Yes	358	4.04	86	15.1	3.71 (2.94, 4.70)	3.58 (2.77, 4.62)	3624	13.5	3.33 (2.99, 3.71)	3.01 (2.69, 3.37)

Rate^#^, incidence rate, per 1,000 person-years; Crude HR*, relative hazard ratio, per 10,000 person-years; Adjusted HR^†^:models including age, sex, comorbidities, and medications.

For all significance: p < 0.001.

### Risks of dyspepsia in patients with or without sleep disturbances stratified by comorbidities

Hazard ratios were significantly elevated for both sleep disorder groups within almost all strata (age, gender, most comorbidities, and with or without sleeping pills) after adjustment (Table 2). Only patients with sleep apnea comorbid with depression, obesity, heart failure, and no benzodiazepine (BZD) use did not have significantly elevated risks of dyspepsia.

### Risks of dyspepsia by subtypes of sleep disorders

Significantly higher risks of dyspepsia were also found for all subtypes of non-apnea disturbance compared to controls (Table 3). Our sensitivity analysis, excluding incident dyspepsia within the 12 months after the index date, supported the robustness of our primary finding.

**Table 3:** Incidence rates and hazard ratios for dyspepsia by subtypes of sleep disorders.

Variables	N	Event	Rate^#^	Crude HR* (95% CI)	Adjusted HR^†^ (95% CI)
**Controls**	90,620	5919	6.73	Reference	Reference
Apnea subgroup	948	175	18.9	2.80 (2.41, 3.25)***	3.65 (3.14, 4.25)***
Non-apnea subgroup					
Insomnia	18,452	3342	20.0	2.92 (2.80, 3.04)***	3.78 (3.60, 3.96)***
Sleep disturbance	10,169	1988	21.9	3.19 (3.03, 3.36)***	4.13 (3.90, 4.37)***
Others	15,741	3202	21.5	3.20 (3.06, 3.34)***	4.29 (4.10, 4.50)***

Rate^#^, incidence rate, per 1,000 person-years; Crude HR*, relative hazard ratio.

Adjusted HR^†^: multivariate analysis including same covariates as Table 2.

### Further analyses on risks of dyspepsia categorized by sleep disturbances and comorbidities

Patients with both sleep disorder and depression had the highest risk of dyspepsia (aHR 5.38, 95% CI 4.93 ~ 5.88), while non-depressed patients with sleep disorder still exhibited a significantly elevated risk of developing dyspepsia (aHR 4.07, 95% CI 3.92 ~ 4.24) (Table 4). Patients that had both sleep disturbance and obesity, heart failure, or no BZD use had higher risks of functional dyspepsia than those with comorbidities but without sleep disturbance (Table 4).

**Table 4:** Further analyses on risks of dyspepsia categorized by with or without sleep disturbances and comorbidities.

Variables		Event	PY	Rate^#^	Adjusted HR^†^ (95% CI)	*p*-value^§^
Sleep disturbance	Depression					< 0.001
No	No	5858	87,595	6.69	Reference	
No	Yes	61	4085	14.9	1.97 (1.53, 2.54)***	
Yes	No	8064	389,898	20.7	4.07 (3.92, 4.24)***	
Yes	Yes	643	25,673	25.1	5.38 (4.93, 5.88)***	
Sleep disturbance	Obesity					0.04
No	No	5895	876,974	6.72	Reference	
No	Yes	24	2606	9.21	1.18 (0.79, 1.77)	
Yes	No	8656	412,637	21.0	4.09 (3.93, 4.26)***	
Yes	Yes	51	2934	17.4	3.20 (2.43, 4.23)***	
Sleep disturbance	Heart failure					0.01
No	No	5824	8,763,062	6.67	Reference	
No	Yes	95	6517	14.6	1.20 (0.98, 1.47)	
Yes	No	8481	408,533	20.8	4.11 (3.95, 4.27)***	
Yes	Yes	226	7038	32.1	3.81 (3.31, 4.39)***	

Rate^#^, incidence rate, per 1000 person-years;

Adjusted HR^†^: mutually adjusted for age, gender, and co-morbidities of diabetes, hypertension, hyperlipidemia, anxiety, peptic ulcer disease, gastroesophageal reflux disease, stroke, ischemic heart disease, obesity, irritable bowel syndrome , cholecystitis , cholelithiasis and other disorders of biliary tract, alcoholism , heart failure , atrial fibrillation , chronic kidney disease , chronic obstructive pulmonary disease, asthma, fatigue and medication of benzodiazepine, zolpidem, aspirin, NSAIDs, steroids, proton pump inhibitors, and H2-receptor antagonistsin Cox proportional hazard regression.

* P < 0.05, ** P < 0.01, *** P < 0.001.

^§^p for interaction.

## Discussion

To our knowledge, this is the first study to investigate the risk of functional dyspepsia associated with sleep disturbances, taking advantage of a population-based dataset derived from a universal health insurance system. We found that risks of functional dyspepsia were significantly higher in patients with sleep disturbances, and that this applied to both the apnea and non-apnea subgroups. These associations remained significantly elevated, following a variety of adjustments and stratifications for demographic factors, comorbidities, and medication use. Further analysis showed that this risk was most elevated in the subgroup of patients with both sleep and depressive disorders.

The associations of interest are consistent with previous cross-sectional and case control studies reporting disrupted or impaired quality of sleep among patients with dyspepsia^11,14,19–21^. However, some studies have suggested stronger associations of dyspepsia with irritable bowel syndrome rather than functional dyspepsia^16–18^ which we did not specifically investigate and would need further research. Our findings help to infer the direction of causation through the prospective design, although the opposite direction of causation (dyspepsia causing sleep disturbance) might also co-explain the cross-sectional relationship and would again need further specific investigation. It is possible that some cases of dyspepsia predate the onset of sleep disturbance despite being diagnosed later; however, this would predict an early divergence in incidence rates that was not sustained over follow-up; this is not apparent in the analysis of cumulative incidence displayed in Fig. 1.

Causal pathways between sleep disturbance and functional dyspepsia remain unclear, but plausible mechanisms include the chronic effects of sleep disturbance on gastrointestinal functions, including hypersensitivity to physiological stimuli, dysfunctions in autonomic nervous regulation, and/or alterations in neuroendocrine responses^20,22^. These will be considered and discussed in the following paragraphs.

First, visceral sensitivity may be altered due to disruptions of circadian rhythm in patients with sleep disturbances resulting in unpleasant abdominal sensations^11–13,16,23^. For example, Zhen et al. described bowel disturbances being significantly higher in rotating shift nurses compared to regular day nurses and that bowel symptom scores correlated with sleep disturbances positively^24^. Previous research has also shown that disruption of Stage 4 or slow-wave (deep) sleep, or the loss of rapid eye movement (REM) sleep, results in hyperalgesia or emergence of abdominal symptoms, such as nausea or cramping in healthy volunteers^15,25–27^, and in patients with functional dyspepsia^12^ or gastroesophageal reflux disease^28^. In addition, poor sleep quality (i.e. increased awakenings or altered sleep stages) has been found to be associated with gastrointestinal symptoms the next day among women with irritable bowel syndrome^29,30^. Other clinical studies have found that melatonin is helpful in reducing pain symptoms of irritable bowel syndrome without affecting colonic motility, and have suggested that this might be due to the restoration of circadian rhythms^16,31^. Although the focus has been more on irritable bowel syndrome than dyspepsia, it is plausible that adverse effects of poor sleep may influence both upper and lower gastrointestinal tracts^10^ and that routine clinical diagnoses obtained from administrative databases may overlap across the two conditions.

Second, gastric motility might be affected by autonomic dysregulation due to reduced sleep efficiency or increased proportions of REM sleep and intrusions during non-REM sleep^10,18,20,32^. These changes may indicate more nocturnal autonomic arousals^20,33^ with similar autonomic effects to waking^20^, as evidenced by increased sympathetic activity^20^, elevated cortisol and adrenocorticotropic hormone, or diminished vagal output^33^, leading to decreased migrating motor complexes in the duodenum^19^. However, since previous research has only described self-reported gastrointestinal discomfort after poor sleep on the preceding night^10,19,20,29^, direct evidence is lacking on whether long-term dysregulation of the autonomic nervous system during sleep might induce visceral motor abnormalities leading to dyspepsia^34^.

Third, sleep disturbance has been found to be associated with alterations in brain regions such as the raphe nuclei, implicated in the release of serotonin, and regulation of circadian rhythm^35^, or the locus coeruleus, implicated in noradrenergic mediated physiological responses to stress and arousal^20,36^. These brain regions also receive afferent neuroendocrine signals from the enteric nervous system mediated by ascending serotonergic, cholinergic, or noradrenergic pathyways^20^, as well as having descending projections that terminate in the dorsal horn to regulate pain sensations. As a neurotransmitter in descending myenteric interneurons, serotonin is responsible for enhancing acetylcholine and calcitonin gene-related peptide release in prokinetic pathways^37^. Interference with such pathways might block the initiation of reflexes within the enteric nervous system and affect gut motility. However, over-stimulation of such pathways would also cause hypersensitivity and bowel discomfort such as nausea, pain, or bloating.

Previous literature on dyspepsia has suggested that comorbidities from other organ systems and possible underlying psychological problems, such as social stressors or depression should be considered as etiological factors^14,21,38,39^. Our findings that the risk of dyspepsia was significantly higher in the sleep disorder groups within almost all comorbidities indicated that, in patients with comorbidities from multiple systems, having sleep disturbance is still key to the risk of their dyspepsia. Our findings of significant associations with sleep disturbance in both people with and without previous diagnoses of depression support the importance of including sleep disturbance alone in considering the etiology of dyspepsia, in addition to the other stressors. The risk of dyspepsia was not as significantly elevated in patients without sleep disturbance, and with only obesity, heart failure, or no BZD use implying that the risk of dyspepsia was influenced much by sleep disturbance than the other conditions. Although further elucidation of underlying mechanisms is still needed, we believe the chronic direct/indirect gastrointestinal effects of sleep disturbance may be implicated, including hypersensitivity to physiological stimuli, and/or dysfunctions in autonomic nervous regulation, and/or alterations in neuroendocrine responses.

Major strengths of this study included the use of a population-based dataset large enough to detect the association of interest, as well as the cohort design with a long observation period, and exposure/outcome measures derived from recorded clinical diagnoses rather than screening instruments. However, there are key limitations which need to be considered in interpreting the findings. First, measurement of comorbid physical disorders was limited to those for which ICD-9-CM codes had been listed, since these diagnoses and records were obtained from a database set up for insurance claims rather than for research purposes (although it is important to bear in mind that recorded diagnoses are required to justify treatments received). The diagnoses were clinician- assigned and might not generalize to research diagnoses. We therefore deliberately chose relatively broad and unambiguous diagnostic groups. Second, although we had access to abundant data on medical service utilizations, information was not present on certain other risk factors associated with dyspepsia, such as dietary habits, smoking^40^, body mass index^10,41^, and psychosocial stressors^39^. There were therefore still some residual confounders and some potential causal pathways that we were not able to explore. Third, the accuracy of the recorded diagnosis of functional dyspepsia needs to be considered, particularly when minor gastrointestinal sensations might be amplified by comorbid mental health conditions. Nevertheless, as we mentioned earlier, all insurance claims from the NHI are scrutinized on a regular basis according to the standardized diagnostic criteria in this study. Although our current study has compensating advantages of naturalistic observations from clinical environment, with regards to the above deficiencies, further well-designed long-term cohorts with diagnoses established by diagnostic interviews or laboratory examinations may help overcome these limitations.

## Conclusions

Using a large, population-based dataset, we found a raised risk of functional dyspepsia in patients with sleep disturbance, both for apnea and non-apnea subgroups. Underlying mechanisms still need further investigation; however, our findings do indicate that sleep disturbance should be identified more systematically and clinical advice/management considered to reduce the risk of functional gastrointestinal disorders.

## Methods

### Data source

Data were analysed from the Longitudinal Health Insurance Database 2000 (LHID 2000), a cohort of one million people (approximately 4.4% of the total population) randomly selected from the Taiwan National Health Insurance Database (NHIRD) from 2000 to 2011.The age and gender distributions of the LHID are not significantly different from Taiwan’s population of 23.75 million^42^. The NHIRD contains and encrypts all healthcare claim data, including diagnostic codes, medical institutions, outpatient and inpatient orders, prescriptions, and expenditures for healthcare from Taiwan’s National Health Insurance (NHI) program launched in 1995: a single-payer insurance program with near-universal coverage of more than 99% of Taiwan’s residents^42^. Personal information in NHIRD cannot be identified after encryption, and use of the NHIRD must be authorized and audited by the Taiwan Bureau of NHI. Diagnoses are coded by physicians according to the International Classification of Diseases, Ninth Revision, Clinical Modification (ICD-9-CM), and are reviewed by the NHI Review Committee every three months to audit the appropriateness of diagnoses and treatments claimed by clinicians. Related to our study, the accuracy and validity of gastrointestinal or ulcer-related diagnoses from the NHIRD have been found to be acceptable when comparing to diagnoses from the medical charts^43^. The study described here was approved by the Institutional Review Board of China Medical University in central Taiwan (CMUH-104-REC2-115). All methods were carried out in accordance with relevant regulations and guidelines. Due to the anonymity of the NHIRD, informed consent is not required from patients providing data.

### Sample ascertainment

For our study cohort, we identified two groups of patients newly diagnosed with sleep disturbances (ICD-9-CM 780.5X), including insomnia or hypersomnia with sleep apnea (780.51, 780.53, 780.57), and non-apnea sleep disorders (307.4X and 780.5X, excluding 780.51, 780.53, and 780.57) between 1998 and 2001. The non-apnea subgroup was further divided into 3 groups: insomnia (780.52), sleep disturbance (780.5X, excluding 780.51, 780.53, and 780.57), and others (sleep disorders or difficulty initiating or maintaining sleep: 307.4; sleep disturbances: 780.50, 780.58 ~ 780.59; other hypersomnia: 780.54; disruptions of sleep–wake cycle: 780.55; or dysfunctions of sleep stages or arousal from sleep: 780.56). The date of the first diagnosis of any of the above sleep disturbances was defined as the index date. Patients with multiple diagnoses of sleep disturbance were included once and assigned to the first index date. Patients with a recorded diagnosis of dyspepsia (536.8) before the index date or for whom age or sex information was incomplete were excluded. The control cohort patients (those without sleep disturbances) were randomly selected from the LHID 2000 and were matched with sleep disturbance cohort patients by age (± 5 years range), gender, and index year on a 1:2 ratio, applying the same exclusion criteria. All exposed and control patients were followed from the index date until the date of receiving a diagnosis of functional dyspepsia, withdrawal from the NHI program, or the end of 2011.

### Outcome and covariates

The primary outcome of our study was dyspepsia (536.8) incorporated from inpatient and outpatient records. Such diagnosis of functional dyspepsia was made by clinicians according to the Rome III and IV criteria: i.e., defining dyspepsia as any combination of four symptoms, including postprandial fullness, early satiety, epigastric pain, and epigastric burning severe enough to affect daily activities and lasted at least 3 days per week over 3 months; with an onset of 6 months in advance. The following baseline recorded diagnoses prior to the index date were also ascertained as comorbidities potentially associated with both sleep disturbance and functional dyspepsia: diabetes (250.XX), hypertension (401.XX ~ 405.XX), hyperlipidemia (272.XX), anxiety (300.00), depression (296.2X ~ 296.3X, 300.4X, 311.XX), cholecystitis (575.XX), cholelithiasis and other disorders of biliary tract (574.XX, 576.XX)^9^, alcoholism (291.XX, 303.XX, 305.00, 305.01, 305.02, 305.03, 790.3X and V11.3)^44^, stroke (430.XX ~ 438.XX), ischemic heart disease (410.XX ~ 414.XX), heart failure (428.XX), atrial fibrillation (427.31), chronic kidney disease (CKD) (580.XX ~ 589.XX), chronic obstructive pulmonary disease (COPD) (491.XX, 492.XX, 496.XX), asthma (493.XX), fatigue (780.79), and obesity (278.XX). For inclusion eligibility (as independent or dependent variables or covariates), any disease diagnosis needed to appear in at least three outpatient visits or one hospitalization. Medication that might be associated with both sleep and dyspepsia was also extracted, namely: benzodiazepines (BZD), zolpidem, aspirin, non-steroidal anti-inflammatory drugs (NSAIDs)^45^, steroids, proton pump inhibitors, and h2-receptor antagonists.

### Statistical analysis

Demographic and clinical characteristics of patients with sleep disturbance diagnoses (apnea subgroup and non-apnea subgroup) and controls were compared using chi-squared tests for categorical variables, and student’s *t*-tests for continuous variables. We computed the incidence rate (per 1000 person-years) of dyspepsia for each cohort. Cox proportional hazards models were used to assess the risk of subsequent dyspepsia associated with exposure status, and hazard ratios (HRs) with 95% confidence intervals (CIs) were estimated. Multivariate models were used to adjust for age, sex, comorbidities, and medication. We also compared the cumulative incidence of dyspepsia between the exposed and control cohorts using the Kaplan–Meier method, and tested their differences by log-rank tests. Additive interactions between comorbidities and sleep disturbance as exposures were also explored. Finally, to reduce the influence of undiagnosed dyspepsia occurring prior to sleep disturbance, a sensitivity analysis excluded dyspepsia in the 12 months after index date. All statistical analyses were performed using SAS (Version 9.3; SAS Institute, Cary, NC, USA) and the significance level was set at 0.05 for two-tailed tests.

## Data Availability

Datasets being analyzed and results being generated and reported in this article can be obtained from the National Health Research Institute of the Ministry of Health and Welfare in Taiwan. Restrictions applied to these data, which were used under license for our study, and so are not publicly available for duplication. Data can be requested only from the Ministry of Health and Welfare.
